# On the round number bias and wisdom of crowds in different response formats for numerical estimation

**DOI:** 10.1038/s41598-022-11900-7

**Published:** 2022-05-17

**Authors:** Hidehito Honda, Rina Kagawa, Masaru Shirasuna

**Affiliations:** 1 Faculty of Psychology, Otemon Gakuin University, 2-1-15, Nishiai, Ibaraki-shi, Osaka, 567-8502 Japan; 2 Faculty of Medicine, University of Tsukuba, 1-1-1 Tennoudai, Tsukuba-shi, Ibaraki, 305-8575 Japan

**Keywords:** Human behaviour, Psychology

## Abstract

When asked for numerical estimations, people can respond by stating their estimates (e.g., writing down a number) or indicating a number on a scale. Although these methods are logically the same, such differences may affect the responses to the numerical estimations. In this study, we examined how differences in response format affected responses to numerical estimations using two behavioral experiments. We found that participants showed a round number bias (i.e., people answered estimates with round numbers) when simply stating a number and the distribution of responses tended to be less diverse. In contrast, this tendency was not observed when the participants responded using a scale. Participants provided more diverse estimates when they answered using a scale. Furthermore, we analyzed how this difference in response distribution was related to the wisdom of crowds (the aggregated judgment is as accurate as, or sometimes better than, the best individual judgment in the group) using computer simulations. The results indicated that round number bias affected the achievement of the wisdom of crowds. Particularly, when the group size was small, biased responses resulted in less effective achievement. Our findings suggest that using an appropriate scale is a low-cost method for eliminating round number bias and efficiently achieving the wisdom of crowds.

## Introduction

Many researchers have examined the psychological processes of numerical estimations in research on judgment and decision-making. For example, previous studies have revealed that people show various biases^1^. For questions such as “What is the height of Mount Everest?” one can respond by stating a number such as “5000 m.” That is, one can describe a number to represent their estimation of the question. How, then, do they choose a number from infinite and countless numbers? The chosen number represents an estimation constructed from knowledge, perception, computation, etc. Several factors may be involved in the estimation process. Among the expected factors, we focused on people’s tendency towards number usage.

Previous studies have shown that people’s psychological processes and behaviors are affected by specific numbers such as round numbers. For example, round numbers play the role of goals, and people’s behaviors are strongly affected by them (professional baseball players have an average batting goal of 0.300, or marathon runners have a goal of 3 or 4 h; these goals strongly affect performance)^2,3^. Round numbers have various effects on communication. People frequently use round numbers in communication^4,5^, and they infer the speaker’s confidence based on whether the speaker uses a round number to communicate numerical information^6,7^.

It is well-known that people tend to use simple strategies such as heuristics in various situations^1^. For example, when judging the frequency of an event, people tend to rely on how easily they can recall the event (i.e., the availability of the event is a heuristic cue in judging frequency). Similarly, in many situations, people may use round numbers as heuristic cues. Behaviors or thinking relying on heuristic cues are sometimes involved with biases^1^. Accordingly, we refer to the tendency to use round numbers as *round number bias*.

The round number bias may affect the responses to the numerical estimations. For example, people tend to use round numbers to respond to numerical estimations. If people show a round number bias in answering estimates, the collected responses may show a biased distribution.

We can examine round number bias by asking for numerical estimations using different methods and comparing them. There are two main methods for obtaining numerical estimations. One is to ask for a response by stating a number such as “Please answer what you think is the height of Mount Everest between 0 and 10,000 m.” The other is to ask for a response by a scale (e.g., presenting a scale with two ends, 0 and 10,000, and asking, “Please answer what you think is the height of Mount Everest between 0 and 10,000 m using this scale”). Although these methods are logically the same, they may affect responses^8–10^. In previous studies, many researchers have conducted works about the effects of different (however, logically the same) information formats on judgment^11,12^. They have also shown that such differences can have various unnoticeable effects in real-world situations^13–15^. Therefore, requiring respondents to answer either numerically or on a scale when asking for numerical estimates may significantly affect numerical estimations. Thus, we believe that comparing numerical estimates obtained using different methods is an important research question for clarifying people’s numerical estimations.

The above issue is an intriguing empirical question about how each method can measure people’s psychological processes of numerical estimations. However, in the present study, we examine this issue in terms of the wisdom of crowds. The wisdom of crowds (referred to as the wisdom of the crowd effect) is when judgments (in the present study, numerical estimations) are made by a group of people, the aggregated judgment (e.g., mean of each judgment) is as accurate as or sometimes better than the best individual judgment in the group^16,17^. Thus far, many researchers have examined how the wisdom of the crowd effect can be achieved from various perspectives^18–28^. In many cases, its discussion is based on the assumption that the opinions of many people are summarized and examined under what conditions it will be achieved well. However, previous studies have also shown that the wisdom of the crowd effect can be achieved with a small number of people (e.g., two or three people)^29–32^. Furthermore, the same effect can be achieved by asking for an opinion several times from a single person and then aggregating them^33–37^. In this study, we focused on the group size to aggregate opinions. In other words, we examined how group size is related to achieving the wisdom of the crowd effect in conjunction with round number bias.

In the next section, we discuss how the round number bias affects the achievement of the wisdom of crowds.

### Round number bias and the wisdom of the crowd effect: effectiveness and efficiency

**Figure 1:**
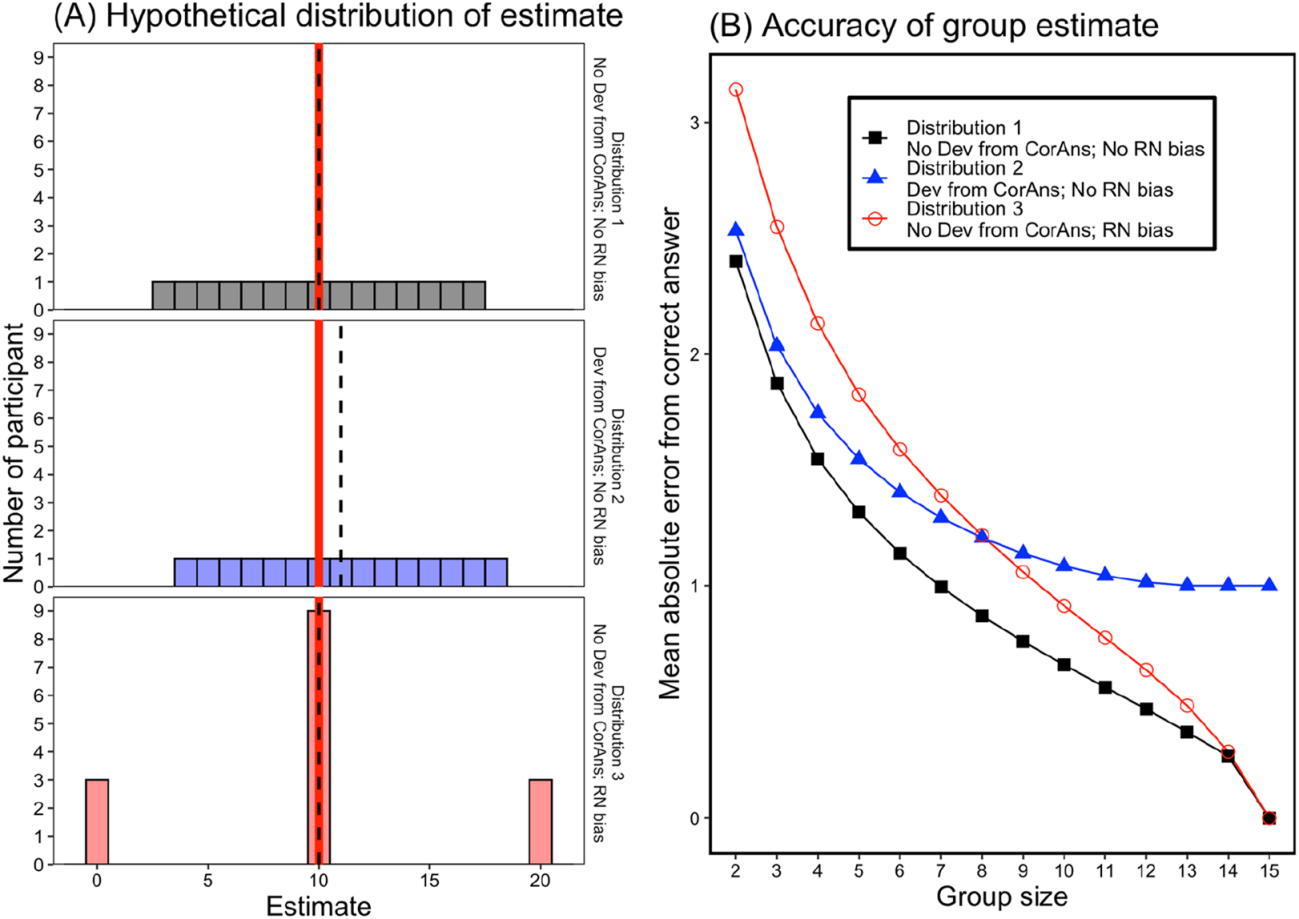
Analysis of possible effects of deviation from the correct answer and round number bias on the wisdom of crowds. (**A**) Three types of responses, based on the two factors—Dev from CorAns (deviation from correct answer) and RN bias (round number bias). The red line indicates the correct value, and the black dashed line indicates the mean for each distribution. (**B**) Accuracy of group estimate (absolute error from correct answer). “Group estimate” indicates the mean of estimates from a group. Since there are many combinations for forming a group, we demonstrate the mean of absolute errors for all possible combinations. For group size 15, since there is one combination for group size 15, the values for groups size 15 indicate the difference between the mean of the distribution and correct answer).

Imagine the following hypothetical situation. Fifteen participants provide numerical estimates. They are asked to estimate between 0 and 20, and the correct answer is 10. Then, imagine the three hypothetical distributions of the estimates, as shown in Fig. 1A. They differ in terms of the existence of deviation from the correct answer (Dev from CorAns, i.e., the mean of the distribution deviates from the correct answer) and the round number bias (RN bias, i.e., participants answer estimates using round numbers). In the top panel (Distribution 1), neither Dev from CorAns nor RN bias exists. By contrast, for the middle (Distribution 2) and lower panels (Distribution 3), Dev from CorAns or RN bias exists. Figure 1B shows the absolute error from the correct answer of the group estimate as a function of the group size for the three distributions. Here, “group estimate” indicates the mean of estimates from a group. We assume that the smaller the absolute error becomes, the better the wisdom of the crowd effect. Since there are many combinations for forming a group, we demonstrate the mean of absolute errors for all possible combinations in Fig. 1B (since there is one combination for group size 15, the values for group size 15 indicate the difference between the mean of the distribution and correct answer).

The two factors, Dev from CorAns and RN bias, are involved in achieving the wisdom of crowds in different ways. The Dev from CorAns relates to the maximum level of error reduction. We refer to this as the *effectiveness* of wisdom of crowds. That is, when Dev from CorAns decreases, the effectiveness of the wisdom of the crowd effect is enhanced. As is apparent in Fig. 1B, the reduction in absolute error differs between Distributions 1 and 2; Distribution 1 reduces to 0 (i.e., the wisdom of the crowd effect is more effective in Distribution 1 than 2). RN bias relates to whether better wisdom of the crowd effects can be achieved with small group size. We refer to this as the *efficiency* of the wisdom of crowds. It is obvious when comparing Distributions 1 and 3. When the group size was smaller than 15, the absolute error of the group estimate in Distribution 1 was lower than Distribution 3. In other words, the wisdom of the crowd effect can be achieved efficiently. Furthermore, note that when the group size is not large enough, group estimates in Distribution 3 (mean of the distribution is in accord with the correct answer) are worse than that in Distribution 2 (the mean of distribution deviates from the correct answer).

Previous studies have discussed how it can be achieved with small group size or even for one person^29–37^. These studies suggest that the wisdom of the crowd effect can be achieved as responses by small group sizes become more diverse. The round number bias is related to this discussion. Accordingly, if round number bias leads to less diverse responses in numerical estimations, it may inhibit the achievement of the wisdom of the crowd effect in a small group size.

This consideration of the effectiveness and efficiency of the wisdom of crowd effect is based on a hypothetical distribution. Therefore, this remains a research question for empirical investigations.

## Current study

Based on the above considerations regarding round number bias and the wisdom of the crowd effect, we examined the following two points. First, we examined whether people would show a round number bias when responding to the numerical estimations. Second, we analyzed how different response formats would affect the wisdom of the crowd effect (the accuracy of the aggregation of obtained estimates). Our specific predictions are summarized as follows:

Regarding the first point, we predicted that people would show a round number bias in responding to numerical estimations. In particular, when asked to respond by stating a number (i.e., the conventional method for eliciting estimates), they would show a strong bias. However, this may not be true when people are asked to respond using the scale. Previous studies have not reported sparse response distributions derived from round number bias when participants were asked to respond using a scale^38^. Hence, we predicted that the round number bias would be attenuated for the scale responses. Here, we also note the bias produced by the scale. Previous studies have discussed response biases produced by a scale^38–47^. For example, people tend to show a central tendency bias (preference for responding around the midpoint rather than endpoints of a scale)^39,40^. Furthermore, a previous study showed that the position of ticks on the scale critically affected responses, and people tended to respond to tick positions^38^. According to these findings, scale design may affect the numerical estimations. However, because this is not the main focus of the present study, we regard this research question (i.e., whether scale design affects numerical estimations) as an explanatory question. We used six scales for explanatory purposes (see the details of the scales in the experimental section).

Regarding the second point, we could make two predictions for the effectiveness and efficiency of the wisdom of crowds based on the expected effect of round number bias. Effectiveness may depend on the relationship between the estimates biased by round numbers and correct answers. When round-biased estimates deviate from the correct answer, the effective wisdom of the crowd effect will not be achieved. In contrast, round-biased estimates become close to correct answers by chance, and effective wisdom of the crowd effect will be achieved. Thus, we predict that there is no general difference in the effectiveness of the wisdom of the crowd effect among response formats. However, the response format critically affects efficiency. When estimates are biased toward round numbers, the good wisdom of the crowd effect may not be achieved with small group size. Thus, we predicted that responses by scale, wherein the round number bias would be attenuated, could more efficiently achieve the wisdom of the crowd effect than those by simply stating a number. Our predictions are summarized as follows:

Prediction (1): People show a round number bias for numerical estimates. This bias depends on the response format. In responding to numerical estimates by stating a number, people show round number bias, and estimates are concentrated on round numbers. However, this bias is attenuated in response to the scale.

Prediction (2): The response format does not affect the effectiveness of the wisdom of crowd effect in general. Responses by stating a number show the lowest (or best) effectiveness depending on problems compared to responses by a scale since biased responses toward round numbers deviate from (or close to) the correct answer.

Prediction (3): The response format critically affects the efficiency of the wisdom of the crowd effect because the round number bias inhibits the achievement of good wisdom of the crowd effect with a small group size. When the group size is small, better wisdom of the crowd effect is achieved in responses by a scale than by stating a number.

**Figure 2:**
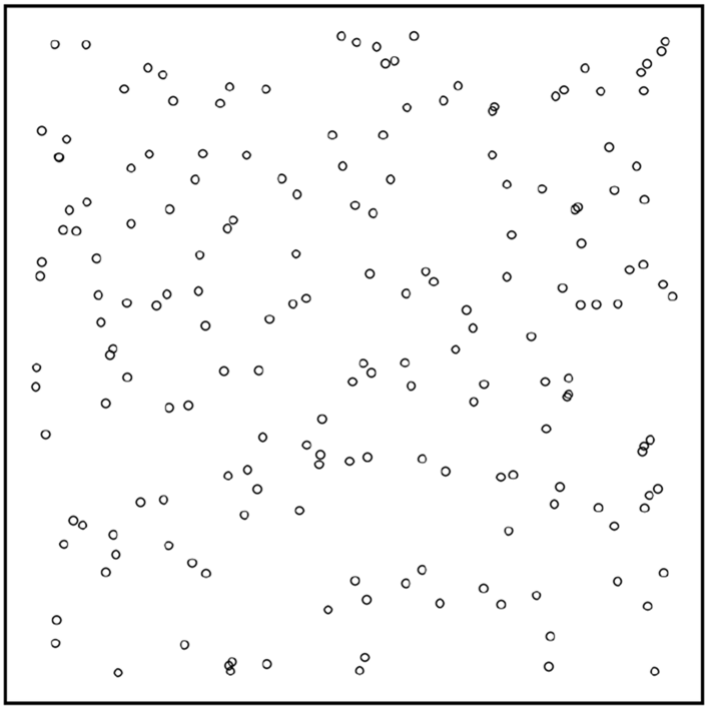
An example of the picture presented in Experiment 1 (random dots estimation task). This is the figure for Question 1 (correct answer: 183).

To examine these predictions, we conducted two behavioral experiments (1,805 Japanese people participated in the two experiments) and computer simulations. The two experiments involved completely different estimation tasks. In Experiment 1 (random-dot estimation task^48^), participants were presented with a picture, as shown in Fig. 2, and asked, “How many dots are in the frame? Please estimate from 0 to 1,000.” In Experiment 2 (general knowledge task), participants were asked to make numerical estimations of general knowledge, such as “What is the elevation of Mont Blanc? Please estimate between 0 and 10,000 m.” We used these tasks for two reasons. First, the nature of the tasks differs. For the random-dot estimation task, nonverbal number sense is expected to be involved in the estimation^50^. For general knowledge tasks, people may rely on numerical induction, heuristics, or specific knowledge^51,52^. We believe that it is important to examine the present issue in different contexts. If round number bias and its related effects are observed in different contexts, we can assume they will be robustly observed.

## Methods

The protocols of the behavioral experiments in the present study conformed to the Declaration of Helsinki and were approved by the Ethics Review Committee for Experimental Research at Otemon Gakuin University. The participants provided online informed consent to participate in the two behavioral experiments.

### Participants

All participants were recruited via Rakuten Insight, a Japanese market research firm (https://insight.rakuten.co.jp/en/). It took about 10–15 min to complete all tasks, and participants received coupons redeemable for online shopping in Japan (about 70 yen, which is around 0.7 US dollars) for their participation.

Detailed information about the participants is presented in Table 1. Regarding the sample size, we took a rather conservative position, since this was the first study to examine how different response formats would affect responses in numerical estimations. The effect size was set as *d* = 0.35. This is between small (*d* = 0.2) and medium (*d* = 0.50) based on the conventional criterion^53^. A power analysis indicated that a sample size of approximately 130 participants per group was required for the study to have 80% power to detect an effect of *d* = 0.35. Based on this analysis, we set the number of participants to 130.

**Table 1:** Number of participants allocated into each group.

	Experiment 1	Experiment 2
Scale 1	128	131
Scale 2	133	127
Scale 3	126	125
Scale 4	126	131
Scale 5	130	128
Scale 6	130	133
Number	128	129
Gender	*n*_*female*_ = 445	*n*_*female*_ = 451
	*n*_*male*_ = 452	*n*_*male*_ = 450
	*n*_*no answer*_ = 4	*n*_*no answer*_ = 3
Age	*M* = 44.48	*M* = 44.70
	*SD* = 8.57	*SD* = 8.44

### Tasks and materials

We used nine and eleven questions in Experiments 1 and 2, respectively. For the two experiments, we set the correct value such that it was equally likely to be located at lower (left side), middle (middle side), or high (right side) values. Regarding the range of responses, we set it from 0 to 1000 in Experiment 1. In Experiment 2, we set the response ranges for the 11 problems based on the results of the preliminary study. Detailed information regarding these materials is provided in the Supplementary Information.

**Figure 3:**
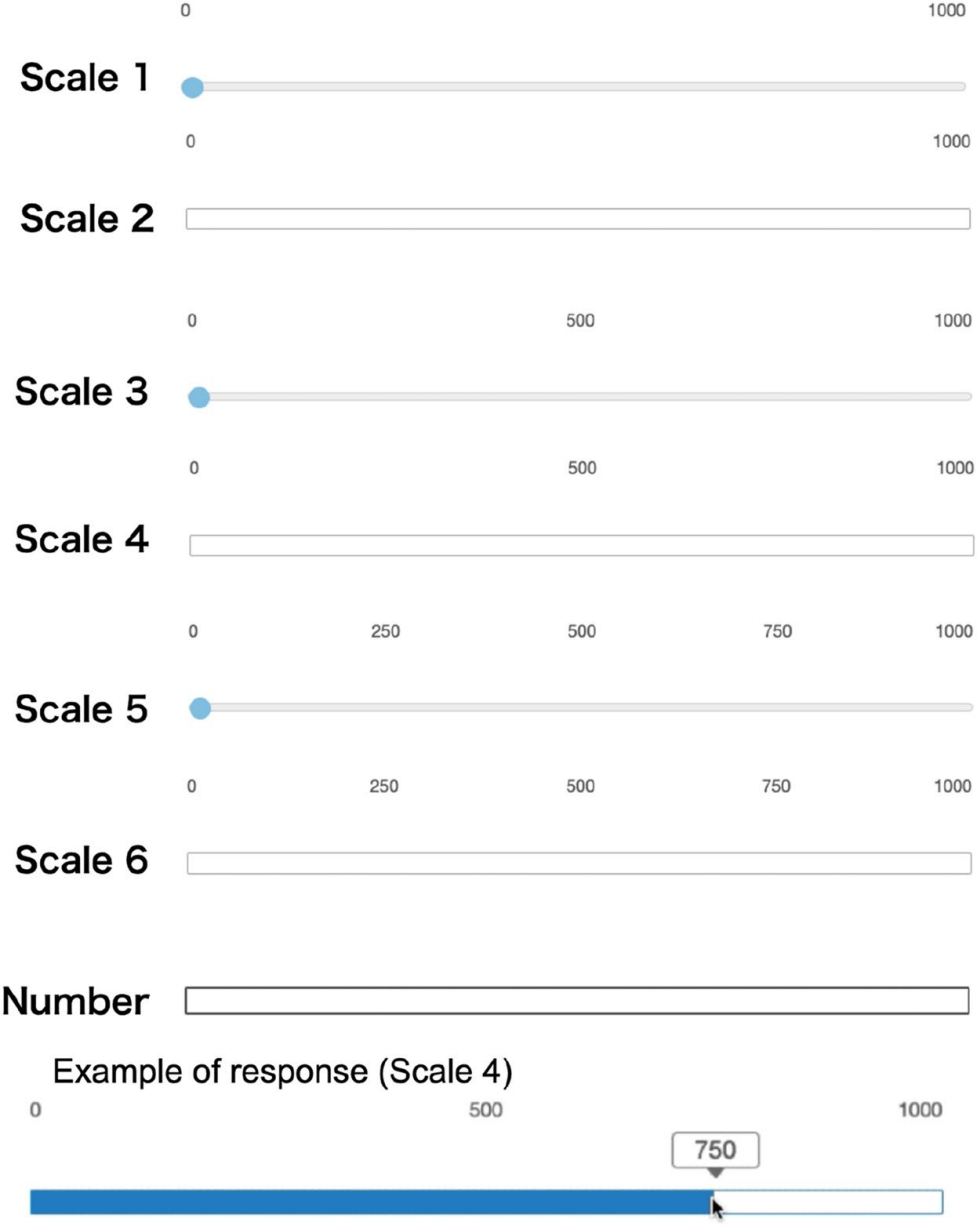
Response format in numerical estimations.

We used Qualtrics (http://www.qualtrics.com) to construct the response format, control the material presentation, and record responses. In responding to numerical estimations with a scale, participants were presented with one of seven response formats, as shown in Fig. 3. The number group wrote the estimates in the box. The scale design differed in terms of the existence of the anchor and number labels on a scale. Regarding the existence of the anchor, on Scales 1, 3, and 5, the anchor value was set to 0, and participants were asked to slide the point to represent their estimates. On Scales 2, 4, and 6, there was no anchor value, and participants were asked to click on the scale representing their estimated value (see the example in Fig. 3). Regarding the differences in the number of labels on the scale, the following differences existed: only the minimum (0) and maximum values displayed (Scales 1 and 2), minimum (0), median, and maximum values displayed (Scales 3 and 4), or quartile values were displayed (Scales 5 and 6).

### Procedure

The procedure for the presentation of the questions was common in both experiments. The presentation order of the questions was randomized for each participant, and participants could answer questions at their own pace. However, the following differences existed between the two experiments. First, in Experiment 2, participants were warned to never look up answers to questions on the Internet, in books, etc., while answering before the experimental session. Second, in Experiment 1, for each question, a random dot picture was presented for five seconds. During the presentation, participants were instructed as follows: “How many dots are in the frame, please estimate from 0 to 1000.” After the 5 s presentation, the picture disappeared, and the participants were asked to provide their estimations.

After each numerical estimation question, participants were asked about their confidence in their responses using a scale labeled “not at all” on the far left and “very confident” on the far right. Confidence ratings were recorded at 101 points. We found that participants were generally not confident about their responses (detailed results are provided in the supplementary document).

## Results

First, we report the participants' comprehension of the task. Using 9 or 11 data points for Experiments 1 and 2, respectively, we calculated the correlation between responses and correct answers for each participant, group, and experiment. If responses and correct answers are positively correlated within a participant [i.e., they answered with a higher (or lower) value when correct answers were high (or low)], we can assume that the participant sufficiently understood the problem and responded appropriately. We found that correlation coefficients were around 0.8 and 0.4 for Experiments 1 and 2, respectively, regardless of response formats. Based on these findings, we can assume that participants understood the task and responded appropriately. More specific summaries are reported in the supplementary documents.

### Response distribution and round number bias in numerical estimations

**Figure 4:**
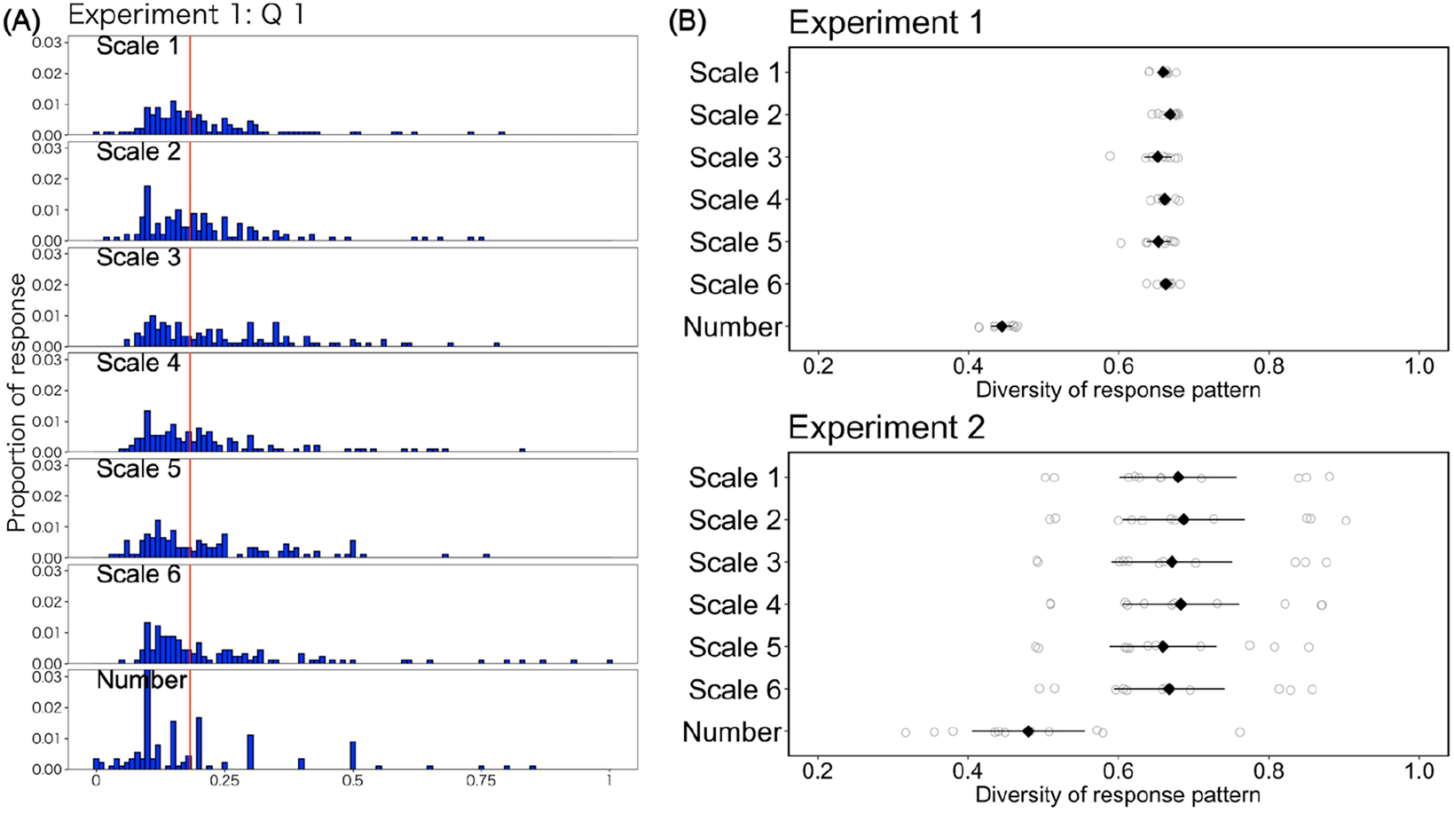
(**A**) Example of response distributions. These histograms were the results for Question 1 in Experiment 1. In making the histogram, each response was rescaled onto a 0 (minimum value in response) to 1 (maximum value in response) scale with 0.01 for bin width. The red line indicates the correct value. (**B**) Diversity of response patterns. Grey dots demonstrate diversity values for each question (nine questions in Experiment 1 and 11 questions in Experiment 2). Black dots demonstrate the means of the diversity values in each group, and the error bars show a 95% confidence interval.

First, we examined Prediction (1) regarding round number bias. Figure 4A shows an example of response distribution (Question 1 in Experiment 1). The responses in the number group were concentrated in round numbers. However, this trend was not observed in the other groups, wherein the participants responded to the questions using a scale. We examined the use of round numbers in responding estimate. In Experiment 1, when a participant used a number such as 0, 100, 200, 300, 400, 500, 600, 700, 800, 900, or 1000, we assumed that they answered the estimate with a round number. In Experiment 2, because the range of numerical estimation was different for each problem, we used a different definition of the round number, depending on the problem. For example, for Question 1 (participants were required to answer between 0 and 10,000), we regarded estimates 0, 1000, 2000, 3000, …, 9000, or 10,000 as round numbers. For Question 4 (participants were required to answer between 0 and 500), we regarded estimates 0, 100, 200, 300, 400, or 500 as round numbers. We have described the definitions of the round numbers in detail in the supplementary document. Table 2 summarizes the round number bias. Participants in the number group used round numbers to answer estimates for about half of the questions. In contrast, participants in the other scale groups rarely used round numbers (the medians were 0 or 1). The usage of round numbers was significantly different between the number and scale groups, and there was no difference between scale groups (see the supplementary document about specific statistics).

**Table 2:** The number of uses of round numbers in answering estimates.

Experiment 1 (9 questions)	Scale 1	Scale 2	Scale 3	Scale 4	Scale 5	Scale 6	Number
Mean	1.070	0.617	1.048	0.683	1.246	0.738	5.523
Median	0	0	0	0	0	0	6
SD	1.900	1.369	1.687	1.237	2.000	1.543	2.911

Experiment 2 (11 questions)	Scale 1	Scale 2	Scale 3	Scale 4	Scale 5	Scale 6	Number
Mean	1.206	1.055	1.432	1.153	1.422	1.203	6.527
Median	1	1	1	1	1	1	7
SD	1.407	1.184	1.542	1.243	1.555	1.536	2.525

These results indicate that round number bias would lead to less diverse response patterns in the number group. To examine this tendency quantitatively, we quantified the diversity of the response distributions in terms of the average uncertainty level (entropy):

1 $$Diversity\, of\, reponse= -\sum_{i}P\left({R}_{i}\right)log\left(P\left({R}_{i}\right)\right)$$

where *P*(*R*_*i*_) denotes the observed proportion of the response pattern *R*_*i*_. For example, in the random-dot task, participants were asked to make numerical estimations between 0 and 1000. Thus, the responses comprised 1,001 patterns. In this measure, when each response is observed as equally likely, it has a higher value. By contrast, when responses tend to be concentrated on particular numbers, they have a lower value. We took a logarithm base such that the measure ranged from 0 to 1 (e.g., the base for the random dot task in Experiment 1 was 1,001). We found that participants in the number group generated a less diverse response pattern than those in other groups (Fig. 4B).

Taken together, we found that participants in the number group showed a round number bias in responding to numerical estimations. As a result, the response patterns in the number group were less diverse than those in the other groups. These results support Prediction (1).

### Basic statistics of numerical estimates

**Figure 5:**
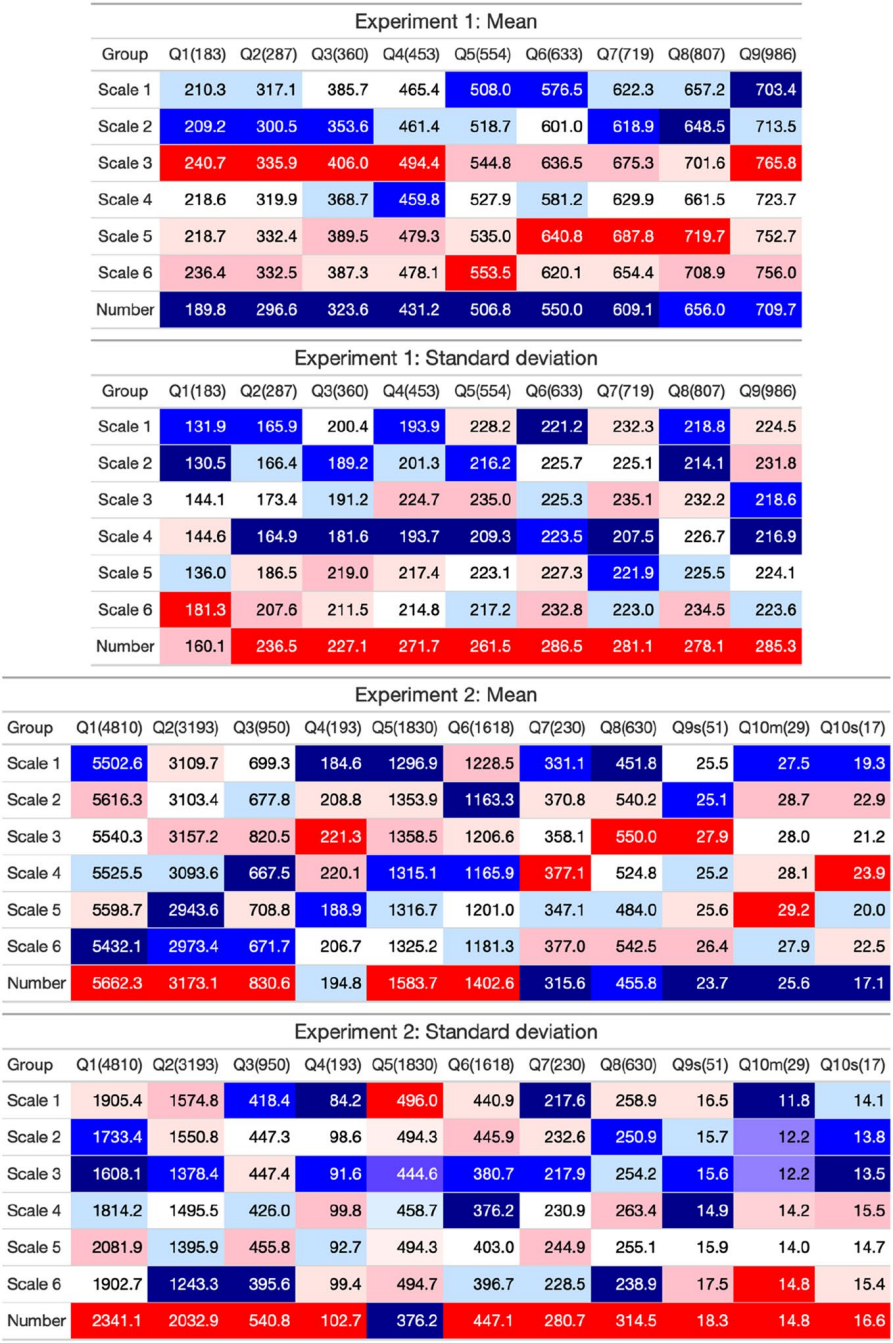
Heatmap of basic statistics (mean and standard deviation) of numerical estimates in Experiments 1 and 2. Each cell is colored based on the order in each question [lowest value (dark blue)-median (white)-highest value (dark red)]. The numbers in the parenthesis next to the question numbers denote the correct answer.

Next, we report the basic statistics of the numerical estimates: the mean and standard deviation (see Fig. 5). As to the mean, although a common tendency in the two experiments was not observed, the mean values in the number group were distinctive. In Experiment 1, the mean values in the number group tended to be lower than those in the other scale groups. In Experiment 2, they were the highest or lowest in 9 out of the 11 questions. Regarding the standard deviation, the standard deviation in the number group tended to be higher than that in the other scale groups. In Experiment 1 (or 2), standard deviations in the number group were highest for eight out of nine (10 out of 11) questions.

**Figure 6:**
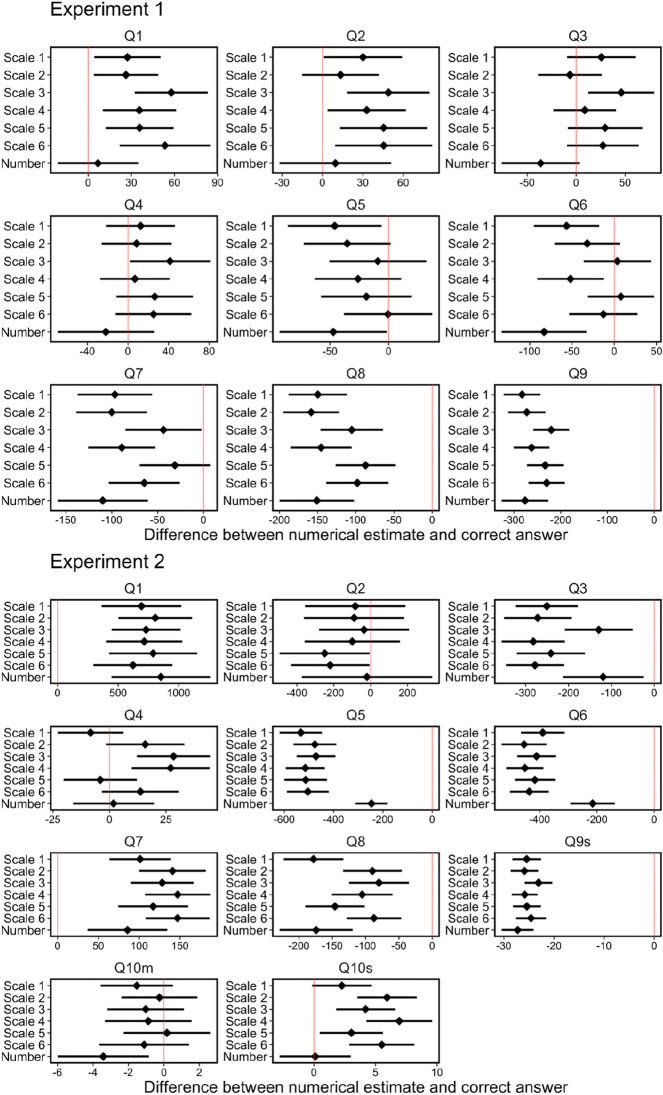
Difference between numerical estimate and correct answer. The point indicates the median, and the error bar indicates a 95% confidence interval. The red line indicates “no deviation” from the correct answer.

How do these characteristics relate to errors in numerical estimations? Next, we examined the accuracy of the estimations in terms of the difference between the estimate and the correct answer. Figure 6 shows the distribution of the differences for each question and group in Experiments 1 and 2. For these data, we examined the accuracy of the group estimates using the following procedure: When the 95% confidence interval of the difference between the estimate and correct answer in a group includes zero, it suggests that the estimates by that group are close to the correct answer. Thus, we counted the number of questions for which the 95% confidence interval included zero. Table 3 summarizes this examination. We did not find an apparent tendency for accuracy, indicating no difference in the accuracy of numerical estimates between the groups.

**Table 3:** Proportion (the number) wherein 95% confidence interval about the difference between an estimate and correct answer contained zero.

Experiment 1						
Scale 1	Scale 2	Scale 3	Scale 4	Scale 5	Scale 6	Number
0.22	0.56	0.22	0.33	0.56	0.44	0.44
2/9	5/9	2/9	3/9	5/9	4/9	4/9

Experiment 2						
Scale 1	Scale 2	Scale 3	Scale 4	Scale 5	Scale 6	Number
0.36	0.27	0.18	0.18	0.18	0.18	0.27
4/11	3/11	2/11	2/11	2/11	2/11	3/11

In summary, the basic statistics varied depending on the response format. However, such a difference did not necessarily lead to a difference in the accuracy of the numerical estimations produced by different answer formats.

### Effect of scale difference on responses

We analyzed the effect of scale differences on responses. According to previous finding^38^, participants may have responded more frequently to numbers described on the scale. To test this possibility, we compared the number of responses that corresponded to the number labels for scales 5 or 6 (i.e., minimum and maximum values, and quartile). That is, if the number label on the scale affects responses, responses that correspond to the number labels for scales 5 and 6 would be more frequently observed for scales 5 and 6 groups than for the other groups.

For each participant, we counted the number of responses that corresponded to the number labels for scales 5 and 6. Table 4 shows the summaries (for specific statistical analyses, see the supplementary document). No apparent trends were observed in Experiment 1. In Experiment 2, it was found that participants in the Scale 5 and 6 groups responded more frequently with scale labeled numbers compared to the other groups. However, the number of responses in the Scale 5 and 6 groups was approximately 1 out of 11 questions on average, suggesting that participants rarely responded with scale-labeled numbers.

**Table 4:** Number of responses that correspond with number labels of Scales 5 and 6.

Experiment 1 (9 questions)	Scale 1	Scale 2	Scale 3	Scale 4	Scale 5	Scale 6
Mean	0.555	0.308	0.675	0.333	0.700	0.546
Median	0	0	0	0	0	0
SD	1.235	0.799	1.192	0.780	1.211	1.208

Experiment 2 (11 questions)	Scale 1	Scale 2	Scale 3	Scale 4	Scale 5	Scale 6
Mean	0.763	0.654	0.976	0.855	1.305	1.271
Median	0.000	0.000	1.000	1.000	1.000	1.000
SD	1.029	0.791	1.160	1.001	1.295	1.447

In summary, the differences in the scales did not strongly affect the responses in the two experiments. In Experiment 2, there was a slight difference in responses due to differences in the scales, and participants in the Scales 5 and 6 groups responded more frequently to the numbers labeled on the scale. However, even if there was an effect, the results indicated that it was not large effect.

### Relationship between response format and the wisdom of crowds

Next, we examined Predictions (2) and (3) using a computer simulation of the wisdom of crowd effect. The computer simulation consisted of three steps.

1. We randomly created groups of sizes 2, 10, 20, 30, 40, or 50 for every seven response format group.
2. For each group and question, we calculated the group estimations. Here, we regarded the mean of the estimations as the group estimation.
3. We calculated the absolute error (the absolute difference between the group estimation and the correct values).

These three steps were repeated 5000 times for each group and question.

**Figure 7:**
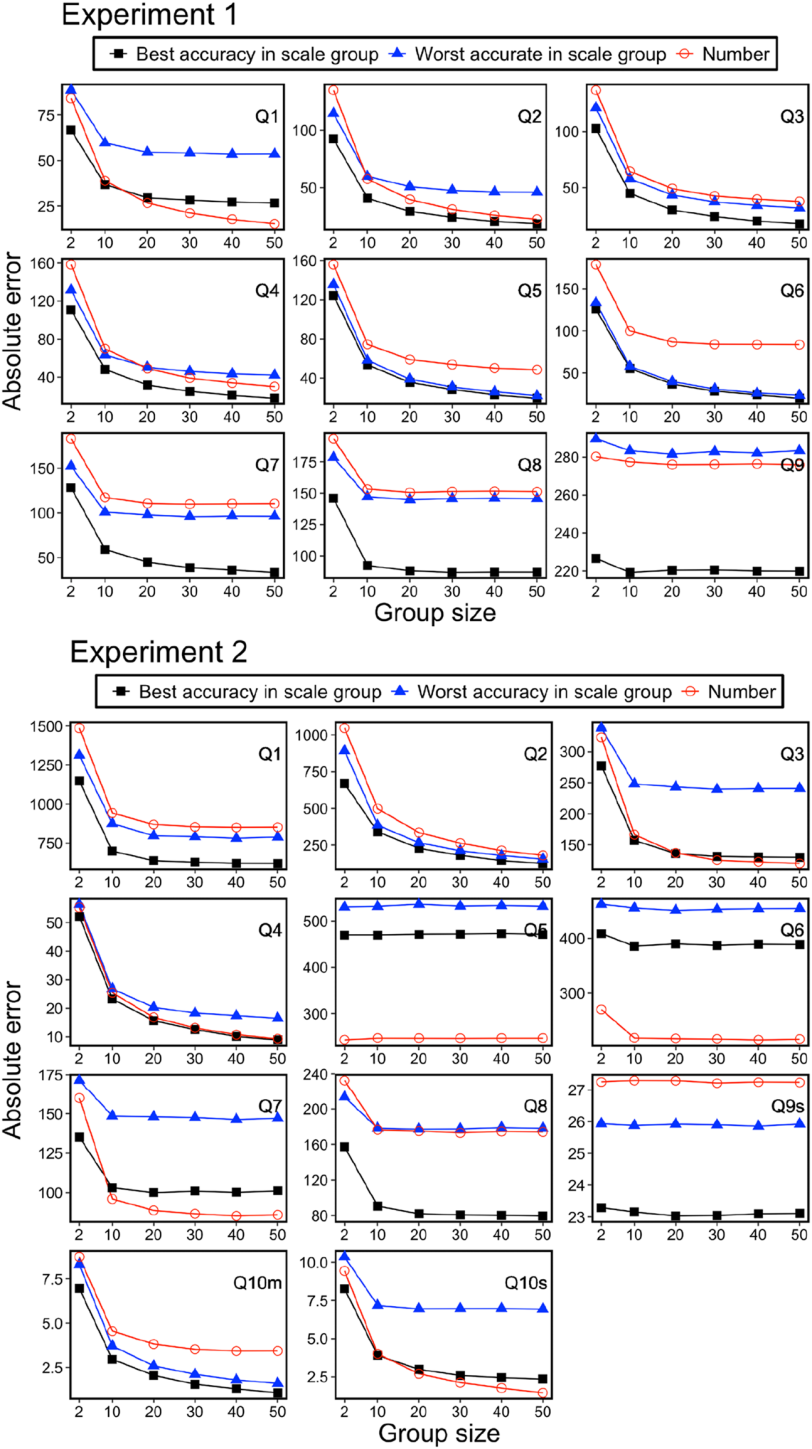
Mean of absolute error (difference between group performances and correct answers) for 5000 computer simulations as a function of group size.

**Figure 8:**
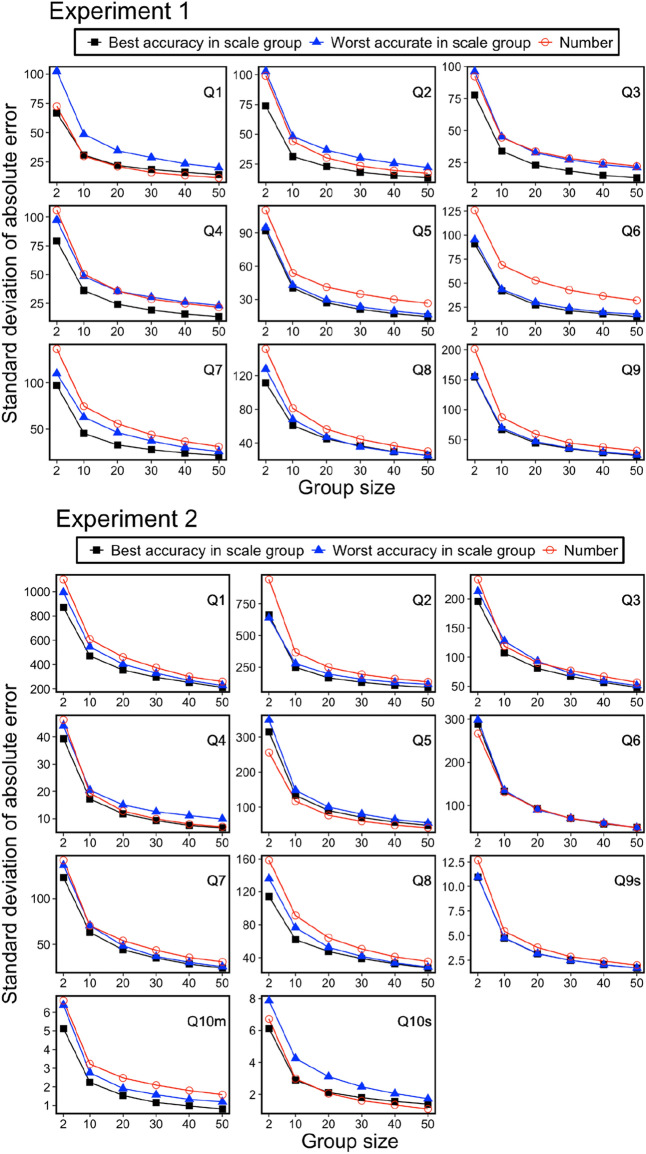
Standard deviation of absolute error (difference between group performances and correct answers) for 5000 computer simulations as a function of group size.

Figures 7 and 8 show the results of the computer simulations. They show the mean and standard deviation of absolute errors, respectively. Since there was no apparent trend in the accuracy of estimate among the six scale groups, we shall report results for the two scales groups, best and worst accuracy scale groups, in the individual estimates for each question. The results indicated four distinctive points. First, the wisdom of the crowd effect was observed in most questions. In other words, the absolute error decreased as the group size increased. Second, as a result of round number bias, participants in the number group tended to show better or worse estimations than those in the scale groups. This result indicated that the group estimation showed good performance (e.g., see Questions 5 and 6 in Experiment 2) when round numbers were concentrated around the correct value by chance. This “by chance” phenomenon also worked adversely: In Experiment 1, participants in the number group tended to show worse estimations than those in the other scale groups. Third, the standard deviation of computer simulation of 5000 group estimations tended to be higher in the Number group than in the other scale groups. In Experiment 1, out of 54 data points (nine questions by six types of group size), the number group showed the highest standard deviations in the 37 data points (68.5%). In Experiment 2, out of 66 data points (11 questions by six types of group size), the number group showed the highest standard deviations in the 40 data points (60.6%). In forming a group, group members may or may not be formed from excellent estimators. The results imply that the number group is more susceptible to the variability of individual differences in the numerical estimations. Fourth and most importantly, the wisdom of the crowd effect was not efficiently achieved in the number group. That is, group performance was relatively worse with a small group size but adequately improved with large group size. As the most pronounced example, see question 2 in experiment 1. The group performance in the number group was the worst among the seven groups when the group size was two. However, when the group size was sufficiently large, the performance improved, and the group performance was comparable with the best accuracy scale group. This kind of reversal phenomenon (here, we defined the “reversal” such that the ordering relationship of the errors at group size 2 does not hold up to group size 50) was observed in three out of nine questions (or four out of eleven questions) in Experiment 1 (or 2).

In summary, the results are generally consistent with Predictions (2) and (3) regarding the wisdom of the crowd effect. When aggregating the estimates of a large number of people, the wisdom of the crowd effect does not differ depending on the response format. In contrast, when aggregating the estimates of a small number of people, the round number bias critically affects the wisdom of the crowd effect. Aggregated estimates by scale responses achieved better wisdom regarding the crowd effect than those by stating numbers. In the next section, we analyze this point in detail using a statistical model.

### Quantitative analyses of the wisdom of crowds

In the preceding section, we showed that the wisdom of the crowd effect depends on the response format and group size. To examine this issue quantitatively, we conducted the following analysis for every question using group performance (here, we defined group performance as the mean of absolute errors in the 5000 simulations). First, for each question, we set the worst performance (the largest absolute error) at the individual level as the benchmark. For example, in Question 4 of Experiment 1, the absolute error at the individual level for the number group was the benchmark for the worst performance. Next, we measured how much wisdom of the crowd effect was achieved for a question *q* in group *g* with a group size of *n* (*achieved wisdom of crowds*, denoted *as AWOC*_*qgn*_) using the following equation:

2 $${AWOC}_{qgn}= 1-\frac{{error}_{qgn}}{{benchmark}_{q}}$$

where *benchmark*_*q*_ is the benchmark in question *q* and *error*_*qgn*_ is the absolute error for question *q*, group *g*, with a group size *n*. *AWOC*_*qgn*_ is between 0 and 1. When *AWOC*_*qgn*_ equals 1 (*error*_*qgn*_ = 0), the complete wisdom of the crowd effect is achieved (the error is eliminated by the aggregation of numerical estimations). By contrast, when *AWOC*_*qgn*_ equals 0 (*error*_*qgn*_ = *benchmark*_*q*_), no wisdom of the crowd effect is obtained (the error is not canceled out by the aggregation of numerical estimations at all). Thus, *AWOC*_*qgn*_ is a quantitative measure of the extent to which the wisdom of crowd effect is achieved.

Using this measure, we analyzed the extent to which the addition of a person to a group would affect the wisdom of the crowd effect in each group. We assume that *AWOC*_*qgn*_ can be described as follows:

3 $${AWOC}_{qgn}= {a}_{qg}\left(1-\frac{1}{1+{b}_{qg}n}\right)$$

In this equation, *a*_*qg*_ (between 0 and 1) represents the maximum level of wisdom of crowds in group *g* for question *q*, indicating the effectiveness of the response format in producing the wisdom of the crowd effect. *b*_*qg*_ (between 0 and 1) represents the contribution of adding a person to the wisdom of the crowd effect in group *g* for question *q*. That is, the maximum level of wisdom of the crowd effect can be achieved with smaller group size, as the *b*_*qg*_ value takes a higher value, indicating the efficiency of the response format for achieving the maximal level of wisdom of the crowd effect. The *b*_*qg*_ representation was based on the idea of a previous study^37^. We estimated the two parameters for each group and problem using a hierarchical Bayesian parameter estimation method and discussed Predictions (2) and (3) based on these two parameters (for the specific procedure, see the Supplementary document).

**Figure 9:**
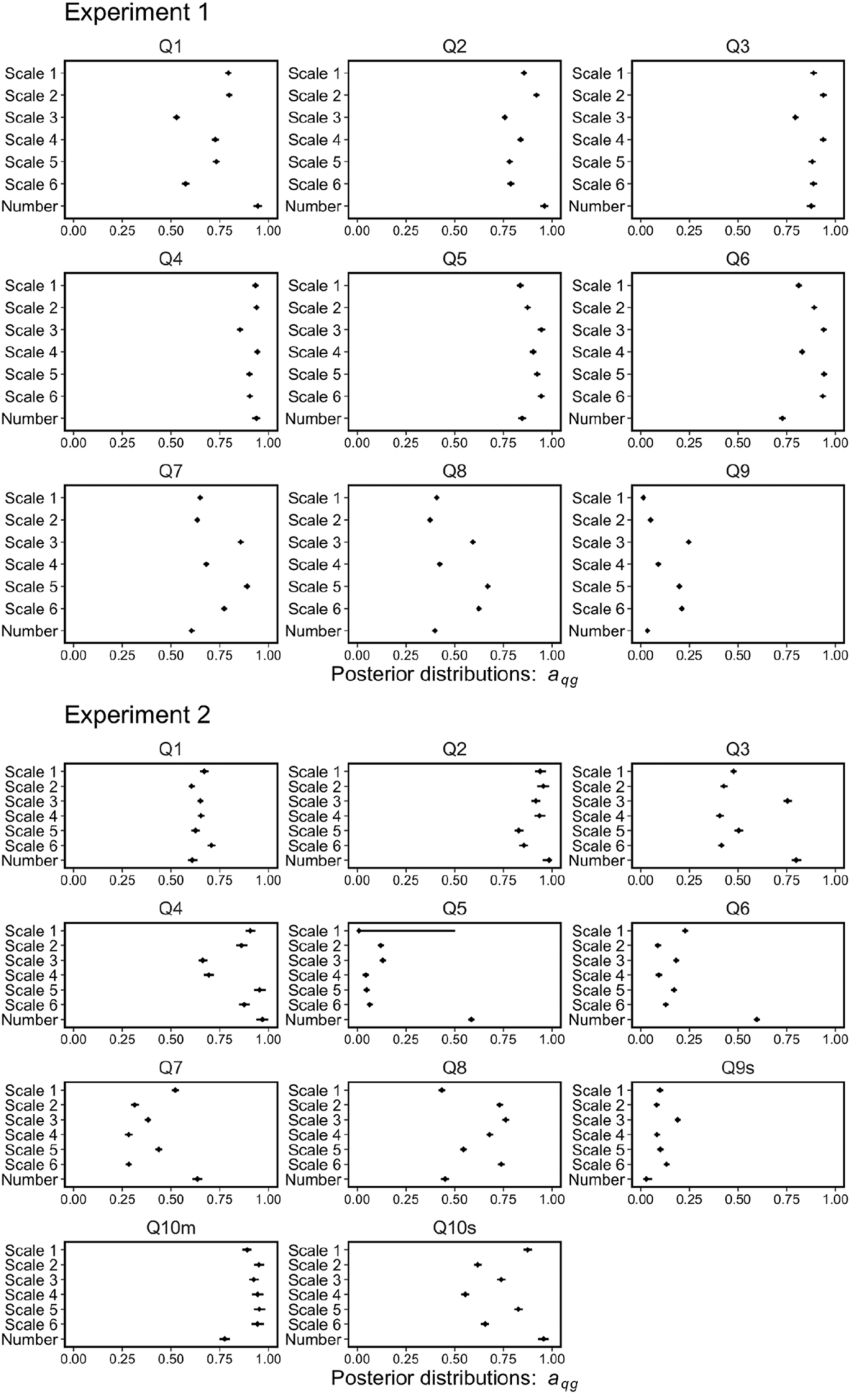
Posterior distributions of the parameters, *a*_*qg*_, in Eq. (3). The point (or error bar) indicates median (or 95% Bayesian credible interval).

Figure 9 shows the posterior distributions of the parameter *a*_*qg*_ in Eq. (3). No common trends were observed. The number group showed the best wisdom of the crowd effect on some problems but also the worst on others. In Experiment 1, among the nine problems, the median of the estimated *a*_*qg*_ was highest in Questions 1 and 2 (or lowest in Questions 6 and 7). In Experiment 2, out of 11 problems, the estimated *a*_*qg*_ was the highest in Questions 2, 3, 4, 5, 6, 7, and 10 s (or lowest in Questions 9 s and 10 m). These results suggest that numerical estimations by the number group had a strong round number bias; as a result, group performance tended to be extreme (best or worst) when the group size was sufficiently large, consistent with Prediction (2) regarding the effectiveness of the wisdom of the crowd effect.

**Figure 10:**
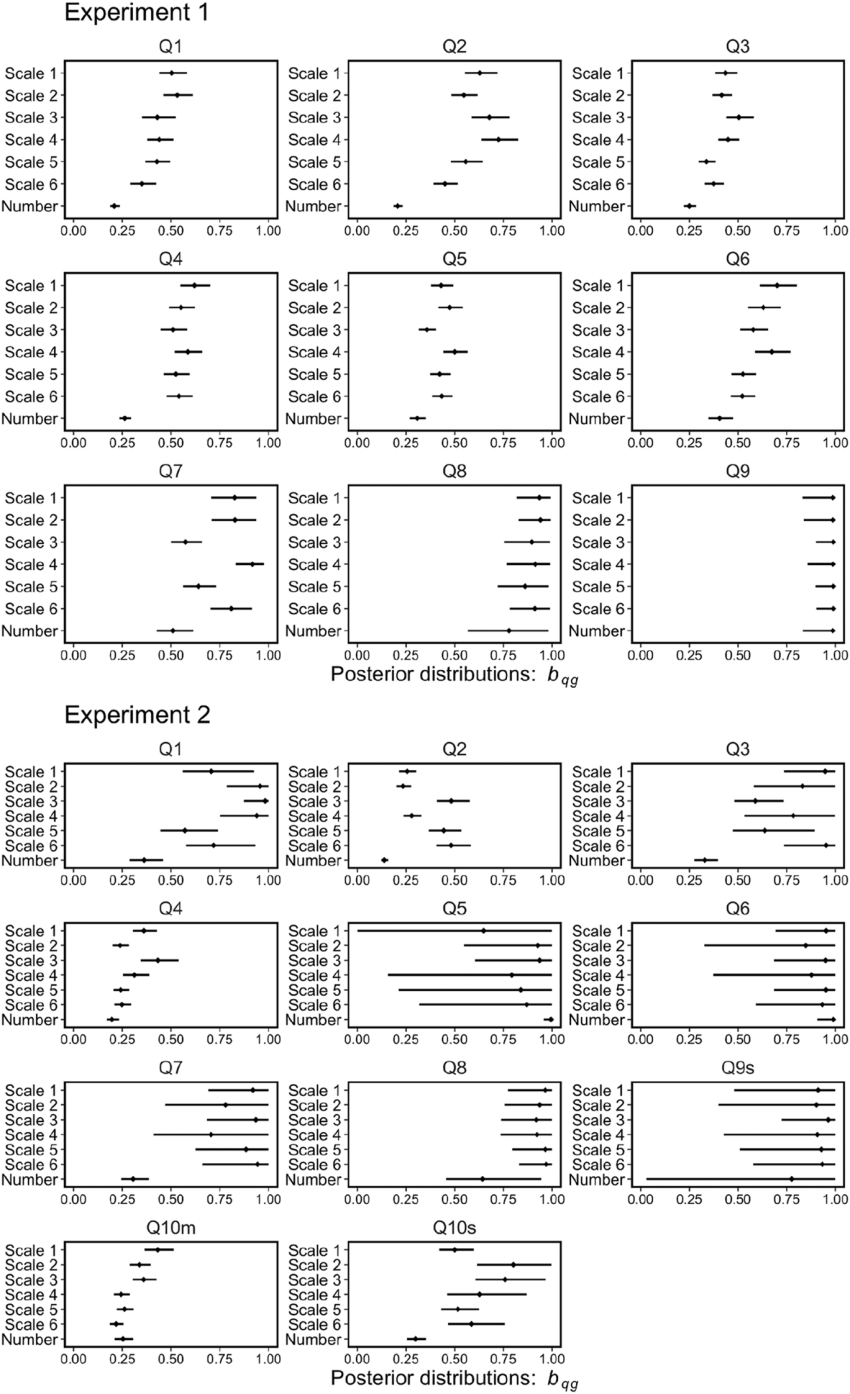
Posterior distributions of the parameters, *b*_*qg*_, in Eq. (3). The point (or error bar) indicates median (or 95% Bayesian credible interval).

**Figure 11:**
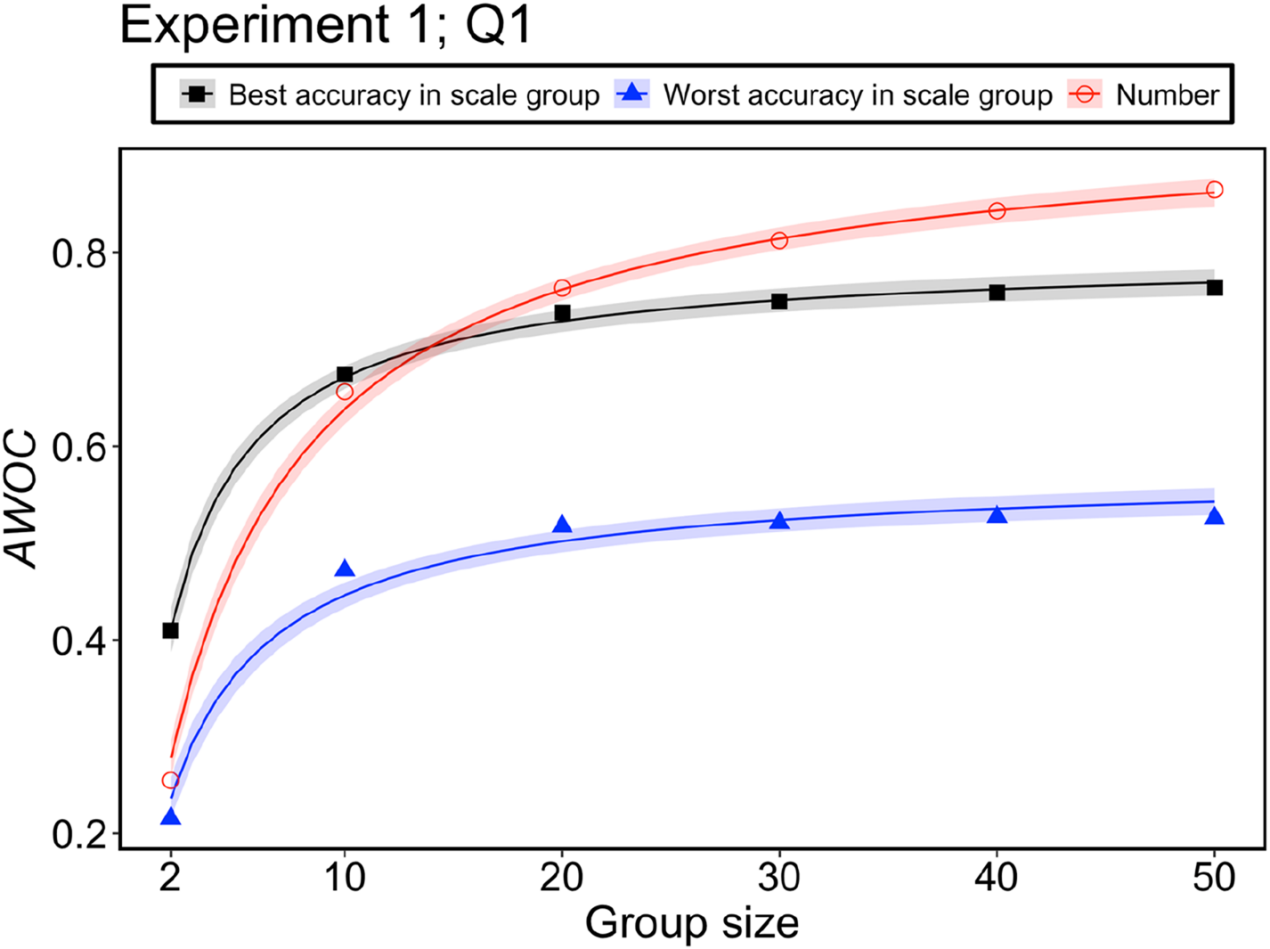
Comparison of *AWOC* between the three groups (the number group and the best and worst accuracy scale groups) in Question 1 of Experiment 1. The point demonstrates the group performance calculated by the computer simulation. The line shows the prediction by the Eq. (3). This prediction was based on the estimated parameters (median of posterior distributions) by the hierarchical Bayesian method. The shadow area indicates a 95% Bayesian credible interval based on estimated parameters in Eq. (3).

The results for the parameter *b*_*qg*_ are noteworthy. Figure 10 demonstrates the posterior distributions of the parameter *b*_*qg*_ in Eq. (3). The posterior distributions for the number group tended to be lower than those of the other groups. In Experiment 1 (or 2), for all problems (or eight out of 11 problems, Questions 1, 2, 3, 4, 7, 8, 9 s, and 10 s), the median estimated *b*_*qg*_ was the lowest. This difference in efficiency for the wisdom of the crowd effect leads to intriguing findings. Figure 11 shows an example of the relationship between *the AWOC* and group size. In this figure, we show the *AWOC*s (points in the figures) calculated from the results of the computer simulations and the predictions (lines and shadow areas in the figures) using Eq. (3). The predictions were calculated using the medians and 95% Bayesian credible intervals of the posterior distributions for the parameters *a*_*qg*_ and *b*_*qg*_. Here, we demonstrated three groups: the number group and the best and worst accuracy groups among the scale groups. The most notable result was that group performance reversed between the number and scale groups as the group size increased. In the example shown in Fig. 11, the number group showed poor performance, as in the worst-scale group with a group size of two. However, the group performance in the number group improved as the group size increased. This outperformed the best group performance in the scale group when the group size exceeded 20. This reversal phenomenon [relationship of group performances between the number group and the worst (or best) scale group was not constant from group size 2 to group size 50] was observed in three out of nine questions in Experiment 1 and five out of 11 questions in Experiment 2. Therefore, the group performance in the number group was comparable with (or sometimes superior to) that in the best-scale group with large group sizes. However, this is not always true for small group sizes.

Together, the findings can be summarized as follows: numerical estimations by the number group did not necessarily result in poor estimations. The number group achieved the highest level of wisdom of crowds effects for some questions. At the same time, the number group achieved the lowest level of wisdom of crowds effect for other questions. Thus, numerical estimations by number group had a round number bias, which led to extreme group performance. This result corroborated Prediction (2). Furthermore, and more importantly, it was found that the wisdom of the crowd effect was not achieved efficiently in the number group compared to the scale group. Thus, the difference in response format critically affected the achievement of the wisdom of the crowd effect, especially when the group size was not sufficiently large, consistent with Prediction (3).

## Discussion

We conducted two behavioral experiments to examine round number bias in answering numerical estimations by comparing different response formats. Additionally, we examined whether the wisdom of the crowd effect depends on the response format using computer simulations. The main findings are summarized as follows. First, when participants responded to numerical estimations by simply stating a number, they showed a round number bias in their responses, and the distributions of their responses became less diverse than those responding to a scale supporting Prediction (1). Second, round number bias affects the achievement of the wisdom of the crowd effect. Depending on the problem, a group whose members responded by numbers achieved better or worse wisdom of the crowd effect than those who responded to numerical estimations by scales, consistent with Prediction (2). Third, the response format critically affected the efficiency of the wisdom of the crowd effect. Due to the round number bias, the number group did not achieve the wisdom of the crowd effect more efficiently than those who responded by scales. This result supports Prediction (3).

We note that the present findings were observed in the different tasks of Experiments 1 and 2 wherein different psychological processes of numerical estimation are expected to be involved. In other words, the present findings are expected to be observed in a wide range of numerical estimation contexts.

To the best of our knowledge, the present study is the first to investigate round number bias in response to numerical estimation. This issue can be investigated by comparing the responses to different response formats. With this comparison, we showed the round number bias in response to numerical estimations by stating a number. The use of round numbers has some communicative functions^6,7^. Thus, the observed round number bias may reflect some communicative functions to achieve adaptive or efficient communication of numerical information. In the present context, wherein we examined the wisdom of the crowd effect with the aggregation of responses, the round number bias adversely affected the efficiency of the wisdom of the crowd effect. However, in different contexts, the round number bias may have different functions. For example, where interaction is involved in tackling a problem^49^, the round number bias may have a communicative function that promotes the wisdom of the crowd effect because people can effectively judge which opinions they should rely on based on whether a round number is used. Thus, further research is necessary to examine the communicative function of round number bias to achieve effective and efficient wisdom of crowd effects from different perspectives.

Although a previous study reported the effects of scale design on responses^38^, we did not find such effects. We have the following thoughts on this difference. First, the design of the scale used in this study was somewhat different from that in a previous study, wherein ticks were displayed on a scale along with numbers^38^. However, this was not the case in this study. This difference may have a strong effect on the responses. Second, the tasks used were completely different, and the effects of different scale designs may depend on the tasks. Since our main focus was the comparison of response patterns between the number and scale formats, we did not examine detailed differences in scales. However, in future research, it is necessary to examine and discuss, in detail, how the differences in scale affect numerical estimations and achievements of the wisdom of crowd effects.

What are the implications of the present findings for real-world application? Based on our findings, when people try to collect opinions from some (“not many”) of their colleagues, friends, or experts around them, they should take care of the response format when asking for numerical opinions. For example, responses by stating a number can indicate opinions that reflect a round number bias. Consequently, the collected opinions may be biased in a specific direction or less diverse. The present findings suggest that using a scale is a low-cost method to eliminate round number bias and collect less biased and more diverse opinions.

Finally, this study has two limitations. First, this study did not examine any variables related to individual differences in cognitive characteristics. Previous studies have shown large individual differences in number sense, and empirical evidence has accumulated from a developmental perspective^50,54^. In future research, it will be necessary to examine individual differences. In particular, examining the interaction between individual differences and response formats will provide intriguing findings. Likewise, cognitive characteristics measured by Cognitive Reflection Test^55^ or Berlin Numeracy Test^56^ may be related to the numerical estimations in the present experimental task. In future research, it will be necessary to examine how these cognitive characteristics affect numerical estimations. These points will also provide interesting insights for analyzing the wisdom of crowd effects. For example, examining the relationship between group members’ cognitive characteristics and the wisdom of the crowd effect will provide insightful evidence for research on the wisdom of the crowd effect.

The second limitation concerns experimental control. In this study, we conducted two web-based experiments. For example, although we warned participants not to look up answers to questions in Experiment 2, they could have, which may have affected the results of the study. Other problem with web-based experiments is the possibility of a low involvement of participants in responses. This may result in undesirable responses, such as "satisfice" (participant may respond with the first minimally acceptable answer that comes to mind without attempting to find an optimal answer)^57^. It is necessary for future research to conduct experiments using different methods (e.g., laboratory experiments instead of web-based experiments) or make manipulations for reducing "satisfice" by devising incentives for participants.

In conclusion, we found that different response formats in numerical estimations generated different response patterns. When participants responded to numerical estimations by stating a number, their response patterns showed round number bias and became less diverse than those on a scale. Additionally, it was found that the round number bias affected the efficiency of the wisdom of the crowd effect. We believe that the present findings provide new evidence about people’s biases in responding to numerical estimations and make substantial contributions from a novel perspective toward understanding how to achieve effective and efficient crowd wisdom.

## Supplementary Information

Supplementary Information.

## Data Availability

The datasets presented in this study can be found in online repositories at https://osf.io/sxdf9/.
