# Supplementary material for: On the round number bias and wisdom of crowds in different response formats for numerical estimation
Source: Sci Rep. 2022 May 17;12:8167. doi: 10.1038/s41598-022-11900-7 (PMC9114128; doi:10.1038/s41598-022-11900-7)
Supplement: Supplementary file 1 — Supplementary Information. [file 41598_2022_11900_MOESM1_ESM.docx]

**Supplementary document**

Various information related to this study can be accessed at https://osf.io/sxdf9/.

**Experimental data**

The data for the two cognitive experiments can be accessed at the directory, “Data.”

**Experimental stimuli**

The random dots pictures presented in Experiment 1 can be accessed at the directory, “stimuli.” The correct values for each image are as follows:

|  | Correct value |
| --- | --- |
| Question 1 | 183 |
| Question 2 | 287 |
| Question 3 | 360 |
| Question 4 | 453 |
| Question 5 | 554 |
| Question 6 | 633 |
| Question 7 | 719 |
| Question 8 | 807 |
| Question 9 | 986 |

**Numbers labels on the scale**

The followings are the summaries of numbers labels on the scale.

Experiment 1 (these are common for all questions)

| Scale type | Numbers labels on the scale |
| --- | --- |
| Scales 1and 2 | 0, 1000 |
| Scales 3 and 4 | 0, 500, 1000 |
| Scales 5 and 6 | 0, 250, 500, 750, 1000 |

Experiment 2

| Scale type | Question | Numbers labels on the scale |
| --- | --- | --- |
| Scales 1and 2 | 1, 2 | 0, 10000 |
| Scales 3 and 4 |  | 0, 5000, 10000 |
| Scales 5 and 6 |  | 0, 2500, 5000, 7500, 10000 |
| Scales 1and 2 | 3 | 0, 1800 |
| Scales 3 and 4 |  | 0, 900, 1800 |
| Scales 5 and 6 |  | 0, 450, 900, 1350, 1800 |
| Scales 1and 2 | 4 | 0, 500 |
| Scales 3 and 4 |  | 0, 250, 500 |
| Scales 5 and 6 |  | 0, 125, 250, 375, 500 |
| Scales 1and 2 | 5, 6 | 0, 2000 |
| Scales 3 and 4 |  | 0, 1000, 2000 |
| Scales 5 and 6 |  | 0, 500, 1000, 1500, 2000 |
| Scales 1and 2 | 7, 8 | 0, 1000 |
| Scales 3 and 4 |  | 0, 500, 1000 |
| Scales 5 and 6 |  | 0, 250, 500, 750, 1000 |
| Scales 1and 2 | 9s, 10m, 10s | 0, 60 |
| Scales 3 and 4 |  | 0, 30, 60 |
| Scales 5 and 6 |  | 0, 15, 30, 45, 60 |

The questions of the general knowledge task in Experiment 2 were as follows.

|  | Content of question | Correct value | Upper limit of response |
| --- | --- | --- | --- |
| Question 1  Question 2  Question 3  Question 4  Question 5  Question 6  Question 7  Question 8  Question 9s  Question  10m, 10s | What is the elevation of Mont Blanc?  What is the elevation of Mount Kitadake?  What is the total number of individuals and organizations that have received the Nobel Prize?  How many countries are members of the United Nations?  In what year did the Tempo Reforms begin?  In what year did the Thirty Years' War in Europe begin?  How many tens of thousands is the population of Nagoya City?  How many tens of thousands is the population of Rio de Janeiro?  What is the world record for the men's 200-meter butterfly in minutes and seconds?  What is the world record (outdoor) for the women's 10,000 meters in minutes and seconds? | 4810  3193  950  193  1830  1618  230  630  51  29(m)  17(s) | 10000  10000  1800  500  2000  2000  1000  1000  60  60(m)  60(s) |

Note. For Question 9, participants were asked to estimate “minute” and “second.” The minute scale for this question ranges from 0 to 10. In other words, only 11 were candidates for the response. Thus, we deleted responses to this question (i.e., minutes) because these response distributions were not suitable for the present analyses.

**Distributions of responses in Experiments 1 and 2**

The distribution of responses for each question in Experiments 1 and 2 can be accessed in the directory, “Distribution_Responses.” In the histogram, each response was rescaled onto a 0 (minimum value in response) to 1 (maximum value in response) scale with 0.01 for bin width. The red line indicates the correct values.

**Comprehension test in Experiments 1 and 2**

To calculate the correlation coefficient, we used raw data for Experiment 1. For Experiment 2, since the scales differed depending on the questions, we standardized responses and correct values such that each response or the correct value was rescaled between 0 (the minimum value in the response scale) and 1 (the maximum value in the response scale). These rescaled values were used to calculate the correlation coefficients.

The distribution of correlation coefficients between responses and correct answers for each participant, group, and experiment can be accessed at the directory, “Comprehension test.” The following tables summarize the distribution.

Experiment 1

| Group | Median | 95% confidence interval |
| --- | --- | --- |
| Scale 1 | 0.796 | 0.769-0.823 |
| Scale 2 | 0.800 | 0.769-0.831 |
| Scale 3 | 0.830 | 0.805-0.855 |
| Scale 4 | 0.820 | 0.797-0.843 |
| Scale 5 | 0.822 | 0.797-0.848 |
| Scale 6 | 0.812 | 0.782-0.842 |
| Number | 0.775 | 0.741-0.809 |

Experiment 2

| Group | Median | 95% confidence interval |
| --- | --- | --- |
| Scale 1 | 0.381 | 0.322-0.440 |
| Scale 2 | 0.320 | 0.257-0.384 |
| Scale 3 | 0.388 | 0.328-0.448 |
| Scale 4 | 0.310 | 0.256-0.364 |
| Scale 5 | 0.369 | 0.307-0.431 |
| Scale 6 | 0.349 | 0.293-0.405 |
| Number | 0.445 | 0.391-0.499 |

**Analysis of the round number bias**

The round numbers were defined as 1/10 of the maximum value of the estimation range. However, in some cases, such as in questions 9 and 10 in Experiment 2, the maximum value was not very high. Therefore, we adjusted the definition according to the maximum value of each problem. The specific definitions are as follows:

Experiment 1

| Question | Round numbers |
| --- | --- |
| 1, 2, 3, 4, 5, 6, 7, 8, 9 | 0, 100, 200, 300, 400, 500, 600, 700, 800, 900, 1000 |

Experiment 2

| Question | Round numbers |
| --- | --- |
| 1 | 0, 1000, 2000, 3000, 4000, 5000, 6000, 7000, 8000, 9000, 10000 |
| 2 | 0, 1000, 2000, 3000, 4000, 5000, 6000, 7000, 8000, 9000, 10000 |
| 3 | 0, 100, 200, 300, 400, 500, 600, 700, 800, 900, 1000,  1100, 1200, 1300, 1400, 1500, 1600, 1700, 1800 |
| 4 | 0, 100, 200, 300, 400, 500 |
| 5 | 0, 100, 200, 300, 400, 500, 600, 700, 800, 900, 1000,  1100, 1200, 1300, 1400, 1500, 1600, 1700, 1800, 1900, 2000 |
| 6 | 0, 100, 200, 300, 400, 500, 600, 700, 800, 900, 1000,  1100, 1200, 1300, 1400, 1500, 1600, 1700, 1800, 1900, 2000 |
| 7 | 0, 100, 200, 300, 400, 500, 600, 700, 800, 900, 1000 |
| 8 | 0, 100, 200, 300, 400, 500, 600, 700, 800, 900, 1000 |
| 9s | 0, 10, 20, 30, 40, 50, 60 |
| 10s | 0, 10, 20, 30, 40, 50, 60 |
| 10m | 0, 10, 20, 30, 40, 50, 60 |

The results of the statistical test on the round number usage are as follows: P-values were adjusted using the Bonferroni method, and effect sizes were reported with Cohen’s *D*.

Experiment 1

| Compared pairs | t value | p value (adjusted) | Effect size |
| --- | --- | --- | --- |
| Scale 1-Scale 2 | 2.220 | 0.573 | 0.275 |
| Scale 1-Scale 3 | 0.101 | 1.000 | 0.013 |
| Scale 1-Scale 4 | 1.924 | 1.000 | 0.242 |
| Scale 1-Scale 5 | 0.724 | 1.000 | 0.090 |
| Scale 1-Scale 6 | 1.541 | 1.000 | 0.192 |
| Scale 1-Number | 14.495 | 0.000 | 1.812 |
| Scale 2-Scale 3 | 2.263 | 0.513 | 0.281 |
| Scale 2-Scale 4 | 0.406 | 1.000 | 0.051 |
| Scale 2-Scale 5 | 2.985 | 0.065 | 0.368 |
| Scale 2-Scale 6 | 0.678 | 1.000 | 0.084 |
| Scale 2-Number | 17.532 | 0.000 | 2.171 |
| Scale 3-Scale 4 | 1.959 | 1.000 | 0.247 |
| Scale 3-Scale 5 | 0.857 | 1.000 | 0.107 |
| Scale 3-Scale 6 | 1.531 | 1.000 | 0.191 |
| Scale 3-Number | 14.963 | 0.000 | 1.878 |
| Scale 4-Scale 5 | 2.701 | 0.155 | 0.338 |
| Scale 4-Scale 6 | 0.319 | 1.000 | 0.040 |
| Scale 4-Number | 17.202 | 0.000 | 2.159 |
| Scale 5-Scale 6 | 2.291 | 0.478 | 0.284 |
| Scale 5-Number | 13.775 | 0.000 | 1.715 |
| Scale 6-Number | 16.533 | 0.000 | 2.059 |

Experiment 2

| Compared pairs | t value | p value (adjusted) | Effect size |
| --- | --- | --- | --- |
| Scale 1-Scale 2 | 0.931 | 1.000 | 0.116 |
| Scale 1-Scale 3 | 1.225 | 1.000 | 0.153 |
| Scale 1-Scale 4 | 0.326 | 1.000 | 0.040 |
| Scale 1-Scale 5 | 1.171 | 1.000 | 0.146 |
| Scale 1-Scale 6 | 0.017 | 1.000 | 0.002 |
| Scale 1-Number | 21.030 | 0.000 | 2.608 |
| Scale 2-Scale 3 | 2.179 | 0.636 | 0.274 |
| Scale 2-Scale 4 | 0.645 | 1.000 | 0.080 |
| Scale 2-Scale 5 | 2.118 | 0.739 | 0.265 |
| Scale 2-Scale 6 | 0.867 | 1.000 | 0.108 |
| Scale 2-Number | 22.142 | 0.000 | 2.768 |
| Scale 3-Scale 4 | 1.599 | 1.000 | 0.200 |
| Scale 3-Scale 5 | 0.052 | 1.000 | 0.007 |
| Scale 3-Scale 6 | 1.195 | 1.000 | 0.149 |
| Scale 3-Number | 19.336 | 0.000 | 2.427 |
| Scale 4-Scale 5 | 1.540 | 1.000 | 0.191 |
| Scale 4-Scale 6 | 0.292 | 1.000 | 0.036 |
| Scale 4-Number | 21.823 | 0.000 | 2.707 |
| Scale 5-Scale 6 | 1.144 | 1.000 | 0.142 |
| Scale 5-Number | 19.497 | 0.000 | 2.432 |
| Scale 6-Number | 20.688 | 0.000 | 2.556 |

**Effect of scale difference on responses**

We compared the number of responses corresponding to the number labels for scales 5 or 6. The results of the statistical test on the number of responses are as follows: P-values were adjusted using the Bonferroni method, and effect sizes were reported with Cohen’s *D*.

Experiment 1

| Compared pairs | t value | p value (adjusted) | Effect size |
| --- | --- | --- | --- |
| Scale 1-Scale 2 | 1.921 | 0.837 | 0.238 |
| Scale 1-Scale 3 | 0.787 | 1.000 | 0.099 |
| Scale 1-Scale 4 | 1.705 | 1.000 | 0.214 |
| Scale 1-Scale 5 | 0.954 | 1.000 | 0.119 |
| Scale 1-Scale 6 | 0.056 | 1.000 | 0.007 |
| Scale 2-Scale 3 | 2.918 | 0.057 | 0.363 |
| Scale 2-Scale 4 | 0.255 | 1.000 | 0.032 |
| Scale 2-Scale 5 | 3.102 | 0.032 | 0.383 |
| Scale 2-Scale 6 | 1.887 | 0.903 | 0.233 |
| Scale 3-Scale 4 | 2.689 | 0.115 | 0.339 |
| Scale 3-Scale 5 | 0.169 | 1.000 | 0.021 |
| Scale 3-Scale 6 | 0.856 | 1.000 | 0.107 |
| Scale 4-Scale 5 | 2.870 | 0.067 | 0.359 |
| Scale 4-Scale 6 | 1.669 | 1.000 | 0.209 |
| Scale 5-Scale 6 | 1.025 | 1.000 | 0.127 |

Experiment 2

| Compared pairs | t value | p value (adjusted) | Effect size |
| --- | --- | --- | --- |
| Scale 1-Scale 2 | 0.959 | 1.000 | 0.119 |
| Scale 1-Scale 3 | 1.553 | 1.000 | 0.194 |
| Scale 1-Scale 4 | 0.730 | 1.000 | 0.090 |
| Scale 1-Scale 5 | 3.728 | 0.005 | 0.463 |
| Scale 1-Scale 6 | 3.279 | 0.025 | 0.404 |
| Scale 2-Scale 3 | 2.582 | 0.218 | 0.325 |
| Scale 2-Scale 4 | 1.790 | 1.000 | 0.223 |
| Scale 2-Scale 5 | 4.840 | 0.000 | 0.606 |
| Scale 2-Scale 6 | 4.240 | 0.001 | 0.526 |
| Scale 3-Scale 4 | 0.895 | 1.000 | 0.112 |
| Scale 3-Scale 5 | 2.124 | 0.727 | 0.267 |
| Scale 3-Scale 6 | 1.798 | 1.000 | 0.224 |
| Scale 4-Scale 5 | 3.131 | 0.041 | 0.389 |
| Scale 4-Scale 6 | 2.711 | 0.150 | 0.334 |
| Scale 5-Scale 6 | 0.200 | 1.000 | 0.025 |

**Method of hierarchical Bayesian parameter estimation**

In the analyses, we estimated parameters *a_qg_* and *b_qg_*. Because parameter *a_qg_* quantifies the maximum level of wisdom of crowds for question *q* in group *g*, the range is constrained between 0 and 1. Parameter *b_qg_* can take any value equal to or greater than 0. However, realistically, it is not expected to have a high value. See Figure 7 in the main text. The results indicate that the wisdom of the crowd effect tended not to reach the maximum level with small group size, such as less than 10. Based on these results, it is reasonable to constrain the upper limit of *the b_qg_*. Hence, as in parameter *a_qg_*, the range was constrained *to b_qg_* between 0 and 1.

To facilitate the estimation procedure, based on the proposed method^1-3^, we transformed the parameters *a_qg_* and *b_qg_* into the probit scale, $a_{qg}^{\varphi}= \Theta^{-1}\left( a_{qg} \right)$, $b_{qg}^{\varphi}= \Theta^{-1}\left( b_{qg} \right)$, where $\Theta^{-1}$ is the inverse cumulative distribution function of the standard normal distribution. Since the parameters cover the whole real line on the probit scale, we assumed that question-level normal distributions produce each group-probitized parameter such that $a_{qg}^{\varphi}\sim N\left( \mu^{a_{q}},\sigma^{a_{q}} \right)$ and $b_{qg}^{\varphi}\sim N\left( \mu^{b_{q}},\sigma^{b_{q}} \right)$, where $\mu^{a_{q}}$and $\mu^{b_{q}}$ are the means of parameters *a* and *b* for question *q*, and $\sigma^{a_{q}}$and $\sigma^{b_{q}}$are standard deviations for question *q*. In addition, we assumed that question-level parameters come from whole-level normal distributions, such that $\mu^{a_{q}}\sim N\left( \mu^{a},\sigma^{a} \right)$ and $\mu^{b_{q}}\sim N\left( \mu^{b},\sigma^{b} \right)$. We assigned priors to all the level parameters as follows: For the means, we used a standard normal distribution: $\mu^{a}\sim N\left( 0, 1 \right)$and $\mu^{b}\sim N\left( 0, 1 \right)$. For the standard deviations, we used uninformative uniform distributions: $\sigma^{a}\sim U\left( 0, 10 \right)$and$\sigma^{b}\sim U\left( 0, 10 \right)$.

We developed a hierarchical Bayesian model using Rstan. Stan code is available at the directory, “Rstan.”

Rstan; https://mc-stan.org/users/interfaces/rstan

**Comparison of AWOC between the three groups (the number group and the best and worst groups in the six scale groups) in Experiments 1 and 2.**

The comparison of AWOC between the three groups (the number group and the best and worst groups in the six scale groups) for each question in Experiments 1 and 2 can be accessed at the directory, “Comparison AWOC.”

**Confidence rating**

The distributions of confidence ratings for each question can be accessed in the directory, “Confidence rating.” In each figure, gray dots demonstrate individual ratings, black dots demonstrate the means of the confidence ratings in each group, and the error bars show 95% confidence intervals.

**References**

1. Rouder, J. N. & Lu, J. An introduction to Bayesian hierarchical models with an application in the theory of signal detection. *Psychon. Bull. Rev.* **12**, 573–604 (2005). [10.3758/bf03196750](https://doi.org/10.3758/bf03196750), Pubmed:[16447374](https://www.ncbi.nlm.nih.gov/pubmed/16447374).

2. Wagenmakers, E. J., Lodewyckx, T., Kuriyal, H. & Grasman, R. Bayesian hypothesis testing for psychologists: A tutorial on the Savage–Dickey method. *Cogn. Psychol.* **60**, 158–189 (2010). [10.1016/j.cogpsych.2009.12.001](https://doi.org/10.1016/j.cogpsych.2009.12.001), Pubmed:[20064637](https://www.ncbi.nlm.nih.gov/pubmed/20064637).

3. Nilsson, H., Rieskamp, J. & Wagenmakers, E.-J. Hierarchical Bayesian parameter estimation for cumulative prospect theory. *J. Math. Psychol.* **55**, 84–93 (2011). [10.1016/j.jmp.2010.08.006](https://doi.org/10.1016/j.jmp.2010.08.006).
